# Targeting Apoptotic Activity Against Prostate Cancer Stem Cells

**DOI:** 10.3390/ijms18081648

**Published:** 2017-07-29

**Authors:** Dagmara Jaworska, Ewelina Szliszka

**Affiliations:** Department of Microbiology and Immunology, School of Medicine with the Division of Dentistry in Zabrze, Medical University of Silesia in Katowice, Jordana 19, 41-808 Zabrze, Poland; eszliszka@sum.edu.pl

**Keywords:** prostate cancer stem cells, TRAIL, apoptosis, paclitaxel, cabazitaxel, docetaxel

## Abstract

Numerous data suggest that an increase of cancer stem cells (CSCs) in tumor mass can be the reason for failure of conventional therapies because of their resistance. CD44+/CD24− cells are a putative cancer stem cells subpopulation in prostate cancer. TRAIL (tumor necrosis factor-related apoptosis-inducing ligand) is an activator of apoptosis in tumor cells. However, some tumors are TRAIL-resistant. Cancer cells can be re-sensitized to TRAIL induced apoptosis by a combination of TRAIL and taxanes. The aim of this work was to analyze the enhancement of the anticancer effect of TRAIL by paclitaxel, cabazitaxel and docetaxel in the whole population of PC3 and DU145 prostate cancer cells, but also in CD44+/CD24− prostate cancer stem cells. We examined the apoptotic effect of TRAIL and taxanes using flow cytometry and Annexin-V-PE staining. The co-treatment with taxanes and TRAIL enhanced significantly the apoptosis in CD44+/CD24− cells only in PC3 cell line but not in DU145 cells. We discovered also that taxanes can increase the expression of death receptor TRAIL-R2 in PC3 prostate cancer cells. The results of our study show that treatment with paclitaxel, cabazitaxel and docetaxel is able to enhance the apoptosis induced by TRAIL even in prostate cancer stem cells.

## 1. Introduction

Prostate cancer represents one of the most prevalent cancers diagnosed in men and remains the second leading cause of cancer related deaths in Europe and the United States [1,2,3]. 

In the last decades, prostate cancer research focused on the hypothesis concerning cancer stem cells (CSCs) and their role in the development of prostate cancer. CSCs were described as a small population of cells that are clonogenic and “possess the capacity to self-renew and to cause the heterogeneous lineages of cancer cells that comprise the tumor” [4,5,6,7,8].

Prostate cancer stem cells were characterized by the expression of several markers such as CD24, CD44, CD49f, CD133, CD166, and α2β1 integrins [6,9,10,11,12,13]. However, the ideal combination which could result in distinction of cancer stem cells have not been found yet, because of prostate cancer genetic heterogeneity.

In 2006, Patrawala et al. [14] demonstrated that CD44+ prostate cancer cells have increased metastatic potential, form colonies in soft agar and tumors in NOD/SCID mice. Afterwards, Hurt et al. [9] discovered that CD44+/CD24− prostate cancer cells have the unique ability to grow as nonadherent spheres in serum replacement medium and have the potential to form tumors in NOD/SCID mice. This marker combination let them identify putative prostate cancer stem cells. Next, studies also identified CD44+/CD24− prostate cancer cells in established prostate cancer cell lines and showed that these subpopulations were more invasive, tumorigenic and were able to differentiate into mature tumor cells expressing highly aggressive phenotype [8,15,16,17].

Those stem-like cells appear to be tumor initiators and possess increased resistance to conventional anti-cancer treatment because of their quiescence [7,18]. 

Cancer stem cells with their increased resistance to antitumor agents can also be unaffected by the immune system mechanisms such as the activation of apoptosis by TRAIL (tumor necrosis factor-related apoptosis-inducing ligand). Cells that exhibit this level of resistance could have been able to escape immune surveillance and become the origin of the neoplastic process. 

TRAIL is a type II membrane protein and it is a member of the tumor necrosis factor (TNF) cytokines superfamily. This molecule induces apoptosis upon binding to its death domain-containing transmembrane receptors: TRAIL-R1 (DR4) and TRAIL-R2 (DR5) [19,20,21]. Other TRAIL receptors exist, which are unable to induce apoptosis. TRAIL-R3 and TRAIL-R4 are known as decoy receptor-1 and -2, because they can inhibit TRAIL-induced apoptosis. Presumably, these receptors protect normal cell from apoptosis induced by ligand TRAIL [19,22]. Ligand induces selectively cell death only in cancer cells, showing little or no toxicity against normal cells. Although TRAIL specifically induces cell death in cancer cells, they can still have resistance to TRAIL-mediated cytotoxicity. In this mechanism of insubordination, an increased expression of anti-apoptotic protein or decreased expression of death receptors (DR4 and DR5) is involved [20,23,24].

Many patients with prostate cancer treated with radical prostatectomy or radiotherapy develop advanced disease and will suffer castration-resistant prostate cancer (CRPC) [25]. Presently, therapies for CRPC include systemic drugs and agents targeted at androgen signaling (novel hormonal agents such as abiraterone and enzalutamide). Food and Drug Administration (FDA)-approved chemotherapy available for patients with prostate cancer includes taxanes, a microtubule-stabilizing drugs, especially docetaxel and cabazitaxel. These drugs bring clinical and survival benefits for many patients, however, due to primary or acquired resistance, their disease will eventually continue to progress [26,27,28,29]. 

Much clinical evidence suggests that cancer stem cells existing in the tumor mass may contribute to treatment failure because of increased chemoresistance to conventional anticancer agents [9,30,31,32,33,34]. 

Cancer stem cells are likely to be more resistant to anti-cancer immune surveillance, such as the process of apoptosis induced by TRAIL ligand. The aim of this study was to prove the hypothesis that cancer stem cells (CSCs) present in the population of prostate cancer cells can be responsible for the increased resistance of the tumor for the natural immune system anticancer agents such as ligand TRAIL. The apoptotic effect of TRAIL in combination with taxanes have been tested in the whole population of PC3 and DU145 prostate cancer cells, but also in CD44+/CD24− prostate cancer stem cells subpopulation within both cell lines to examine if this compounds can augment the anti-cancer effect of TRAIL.

## 2. Results

### 2.1. Apoptotic Activity of TRAIL (Tumor Necrosis Factor-Related Apoptosis-Inducing Ligand) in DU145 and PC3 Prostate Cancer Cell Lines

**Figure 1:**
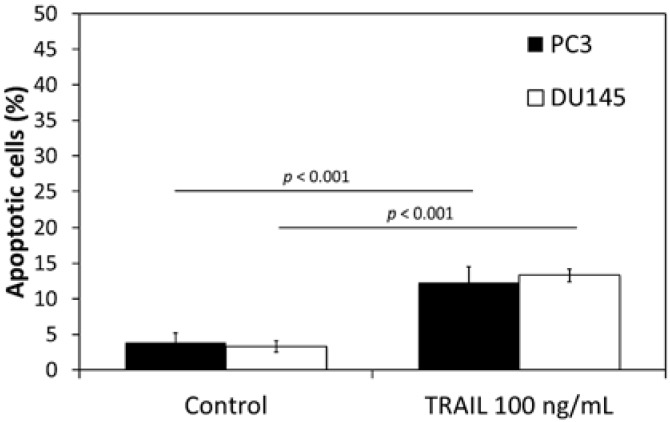
Apoptotic effect of TRAIL (Tumor necrosis factor-related apoptosis-inducing ligand) (100 ng/mL) in DU145 and PC3 prostate cancer cells. The values represent mean ± SD (*n* = 6).

The apoptotic effect of TRAIL at the concentration of 100 ng/mL following a 48 h incubation was 12.3% ± 2.3% of apoptotic cells in PC3 cell line and 13.3% ± 0.9% of apoptotic cells in DU145 cell line. Apoptotic effect analyzed by flow cytometry is presented in Figure 1. Our data confirm that both analyzed cell lines are TRAIL-resistant.

### 2.2. Apoptotic Activity of Paclitaxel, Cabazitaxel or Docetaxel in DU145 and PC3 Prostate Cancer Cell Lines

The apoptotic effect of paclitaxel, cabazitaxel and docetaxel against PC3 and DU145 cells depends on a concentration of the tested compound. In our study, we used tested compounds in the concentrations from 0.1 µM to 1 µM. The most effective concentrations were 0.25 µM and 0.5 µM. Further increasing the concentration of tested compounds does not have a significant effect on apoptosis level. 

**Figure 2:**
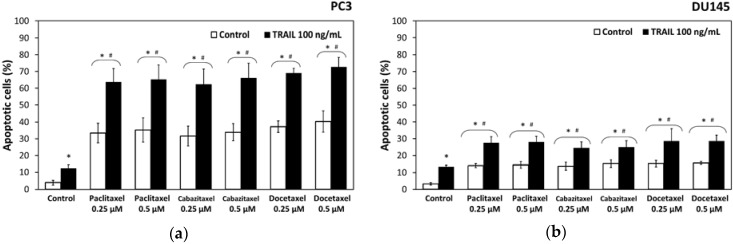
Apoptotic effect of TRAIL (100 ng/mL) in combination with paclitaxel, cabazitaxel and docetaxel in DU145 and PC3 prostate cancer cells: (**a**) apoptotic effect in PC3 cells; and (**b**) apoptotic effect in DU145 cells. * *p* < 0.001, significantly different from the respective control; # *p* < 0.001, significantly different from TRAIL alone. The values represent mean ± SD (*n* = 6).

Paclitaxel induced apoptosis in 33.4% ± 5.8% of cancer cells in a concentration of 0.25 µM and 33.9% ± 5.1% in a concentration of 0.5 µM in PC3 cells after a 48 h incubation. Cabazitaxel induced apoptosis in 31.6% ± 5.9% of cancer cells in a concentration of 0.25 µM and 33.9% ± 5.1% in a concentration of 0.5 µM in PC3 cells. In the same cell line docetaxel induced apoptosis in 37.3% ± 3.4% cells in a concentration of 0.25 µM and 40.3% ± 6.4% in a concentration of 0.5 µM. Apoptotic effects of paclitaxel, cabazitaxel and docetaxel determined by flow cytometry in PC3 cell line are presented in Figure 2a.

In the other tested prostate cancer cell line DU145, paclitaxel induced apoptosis in 14.1% ± 1.2% cells in a concentration of 0.25 µM and 14.4% ± 1.8% in a concentration of 0.5 µM after a 48 h incubation, whereas cabazitaxel induced apoptosis in 13.7 ± 2.3% cells in a concentration of 0.25 µM and 15.3% ± 2.2% in a concentration of 0.5 µM in DU145 cells. Docetaxel induced apoptosis in 15.3% ± 1.90% cells in a concentration of 0.25 µM and 15.6% ± 0.9% in a concentration of 0.5 µM in DU45 prostate cancer cell line. Therefore, DU145 prostate cancer cells were more resistant to apoptotic activity of used taxanes. Apoptotic effects of paclitaxel, cabazitaxel and docetaxel determined by flow cytometry in DU145 cell line are presented in Figure 2b. 

### 2.3. Apoptotic Activity of TRAIL in Combination with Paclitaxel, Cabazitaxel or Docetaxel in DU145 and PC3 Prostate Cancer Cell Lines

The combined treatment of TRAIL and paclitaxel, cabazitaxel or docetaxel significantly increased the apoptotic effect on PC3 and DU145 prostate cancer cells compared to TRAIL or taxane used alone. We examined the apoptotic effect of 100 ng/mL TRAIL in combination with 0.25 µM and 0.5 µM paclitaxel, cabazitaxel or docetaxel against PC3 and DU145 prostate cancer cells. Figure 2 demonstrates the percentage of apoptotic cells stained with Annexin V-PE and analyzed by flow cytometry in PC3 (Figure 2a) and DU145 (Figure 2b). 

Combined treatment with TRAIL and paclitaxel induced apoptosis in 63.4% ± 8.1% of cancer cells in a concentration of 0.25 µM and 65.3% ± 8.7% in a concentration of 0.5 µM in PC3 cells after a 48 h incubation. Cabazitaxel and TRAIL induced apoptosis in 62.3% ± 9.3% of cancer cells in a concentration of 0.25 µM and 66.1% ± 8.8% in a concentration of 0.5 µM in PC3 cells. In the same cell line combination of TRAIL and docetaxel induced apoptosis in 69.0% ± 2.8% cells in a concentration of 0.25 µM and 72.6% ± 5.8% in a concentration of 0.5 µM. 

In the DU145 prostate cancer cell line, co-treatment with paclitaxel and TRAIL induced apoptosis in 27.5% ± 3.8% cells in a concentration of 0.25 µM and 28.0% ± 3.4% in a concentration of 0.5 µM after a 48 h incubation, whereas cabazitaxel and TRAIL induced apoptosis in 24.5% ± 3.75% cells in a concentration of 0.25 µM and 24.9% ± 3.9% in a concentration of 0.5 µM in DU145 cells. Docetaxel and TRAIL co-treatment induced apoptosis in 28.6% ± 7.35% cells in a concentration of 0.25 µM and 28.6% ± 3.5% in a concentration of 0.5 µM in DU45 prostate cancer cell line. DU145 prostate cancer cells were more resistant to TRAIL and taxanes activity compared to PC3 cells.

The data indicate that paclitaxel, cabazitaxel and docetaxel augment the apoptotic activity of TRAIL against both prostate cancer cell lines and sensitize these TRAIL-resistant cells to apoptotic activity of TRAIL.

### 2.4. Necrotic Effect of TRAIL and/or Paclitaxel, Cabazitaxel, Docetaxel in DU145 and PC3 Prostate Cancer Cell Lines

The necrotic cell death percentage in PC3 and DU145 cells treated with paclitaxel, cabazitaxel and docetaxel examined by LDH assay was almost in all cases not significant compared to the control. In the flow cytometry test with 7-AAD, necrotic effect of TRAIL and/or paclitaxel, cabazitaxel and docetaxel in PC3 and DU145 cells was not significant compared to control. Therefore, in this test, no significant necrotic effect was shown in PC3 and DU145 prostate cancer cell lines.

### 2.5. Cancer Stem Cells in DU145 and PC3 Prostate Cancer Cell Lines

Using flow cytometry we identified CD44+/CD24− subpopulation in PC3 and DU145 human prostate cancer cell lines. As Hurt et al. reported, it is a small subpopulation in prostate cancer cell lines with increased clonogenic properties, tumorigenic capacity and stem-like characteristics [9]. Therefore in this paper, we used abbreviation CSCs for the CD44+/CD24− subpopulation and non-CSCs for the CD44+/CD24− depleted subpopulation.

**Figure 3:**
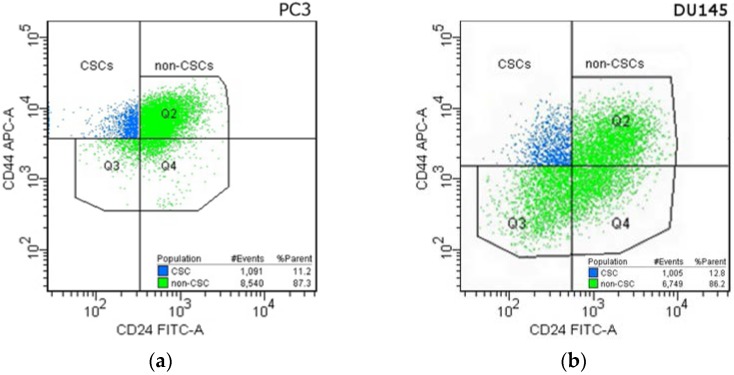
Cytometric analysis of Cancer Stem Cells in: PC3 (**a**) and DU145 (**b**) prostate cancer cell lines. Cells were incubated with anti-CD24 FITC-labeled and anti-CD44 APC-labeled antibodies. CD44+/CD24− population marked as blue (CSCs), the CD44+/CD24− depleted population marked as green (non-CSCs). Gates were established based on the appropriate isotype controls.

In the PC3 cell line, the CSCs subpopulation was 11.4% ± 1.9%, whereas, in the DU145 cell line, 12.6% ± 0.9%. Figure 3 presents subpopulations gated in the PC3 (Figure 3a) and DU145 (Figure 3b) prostate cancer cells. CSCs subpopulation was marked blue, non-CSCs subpopulation was marked green.

### 2.6. Apoptotic Activities of TRAIL within the Subpopulations in DU145 and PC3 Prostate Cancer Cell Lines

Apoptosis was measured by flow cytometry using the Apoptosis-PE Kit with Annexin V, anti-CD24-FITC and anti-CD44-APC monoclonal antibodies were used simultaneously to distinguish CSCs and non-CSCs subpopulation. The CSCs and non-CSCs subpopulation were gated in the FACS DIVA software (Figure 3), and for each population the apoptotic cells number was measured according to Annexin V-PE expression.

**Figure 4:**
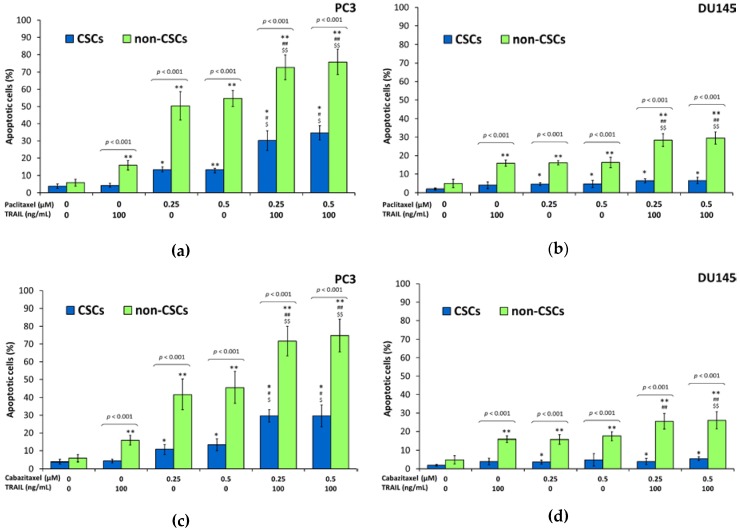

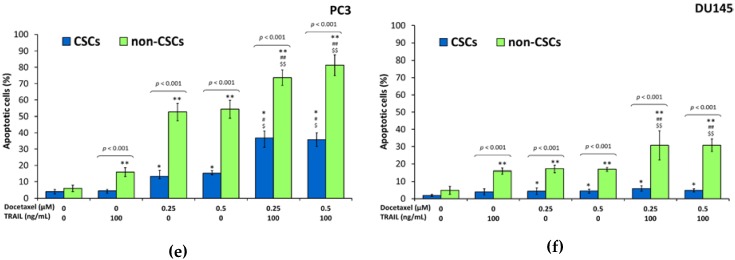
Apoptotic effect of TRAIL (100 ng/mL) in combination with paclitaxel, cabazitaxel and docetaxel in CSCs and non-CSCs subpopulations within DU145 and PC3 prostate cancer cells. The values represent mean ± SD (*n* = 6). Within CSCs subpopulation: * *p* < 0.05, significantly different from the respective control; # *p* < 0.05, significantly different from TRAIL alone. $ *p* < 0.05, significantly different from respective taxane alone. Within non-CSCs subpopulation: ** *p* < 0.05, significantly different from the respective control; ## *p* < 0.05, significantly different from TRAIL alone. $$ *p* < 0.05, significantly different from respective taxane alone. (**a**) Apoptotic effect of TRAIL (100 ng/mL) in combination with paclitaxel, within CSCs and non-CSCs subpopulations in PC3 prostate cancer cells; (**b**) Apoptotic effect of TRAIL (100 ng/mL) in combination with paclitaxel, within CSCs and non-CSCs subpopulations in DU145 prostate cancer cells; (**c**) Apoptotic effect of TRAIL (100 ng/mL) in combination with cabazitaxel, within CSCs and non-CSCs subpopulations in PC3 prostate cancer cells; (**d**) Apoptotic effect of TRAIL (100 ng/mL) in combination with cabazitaxel, within CSCs and non-CSCs subpopulations in DU145 prostate cancer cells; (**e**) Apoptotic effect of TRAIL (100 ng/mL) in combination with docetaxel, within CSCs and non-CSCs subpopulations in PC3 prostate cancer cells; (**f**) Apoptotic effect of TRAIL (100 ng/mL) in combination with docetaxel, within CSCs and non-CSCs subpopulations in DU145 prostate cancer cells.

**Figure 5:**
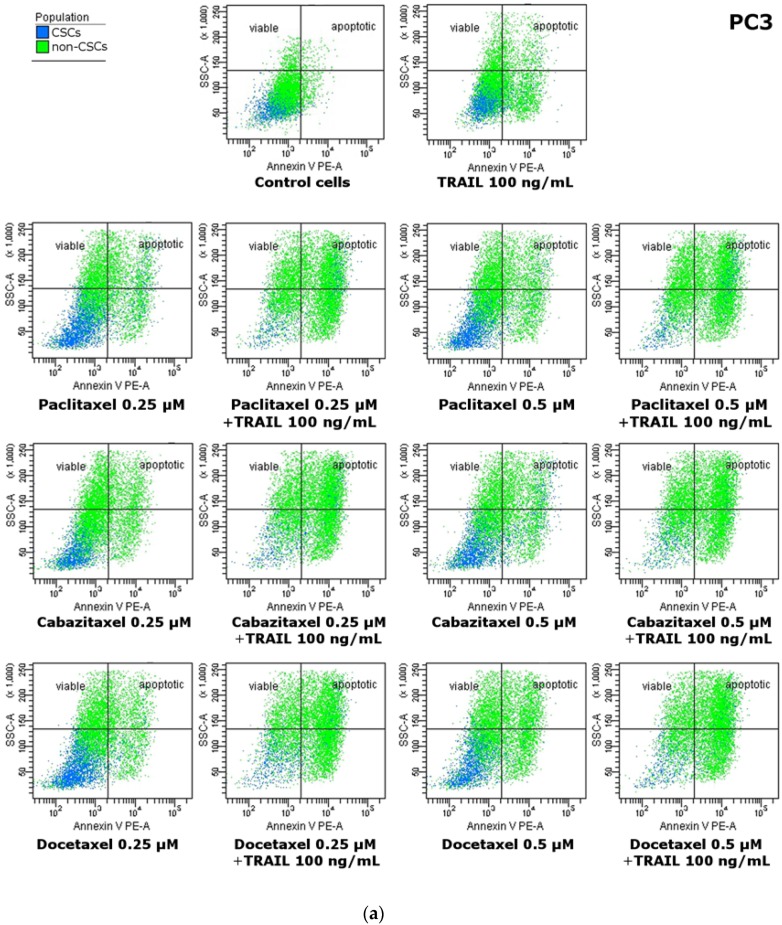

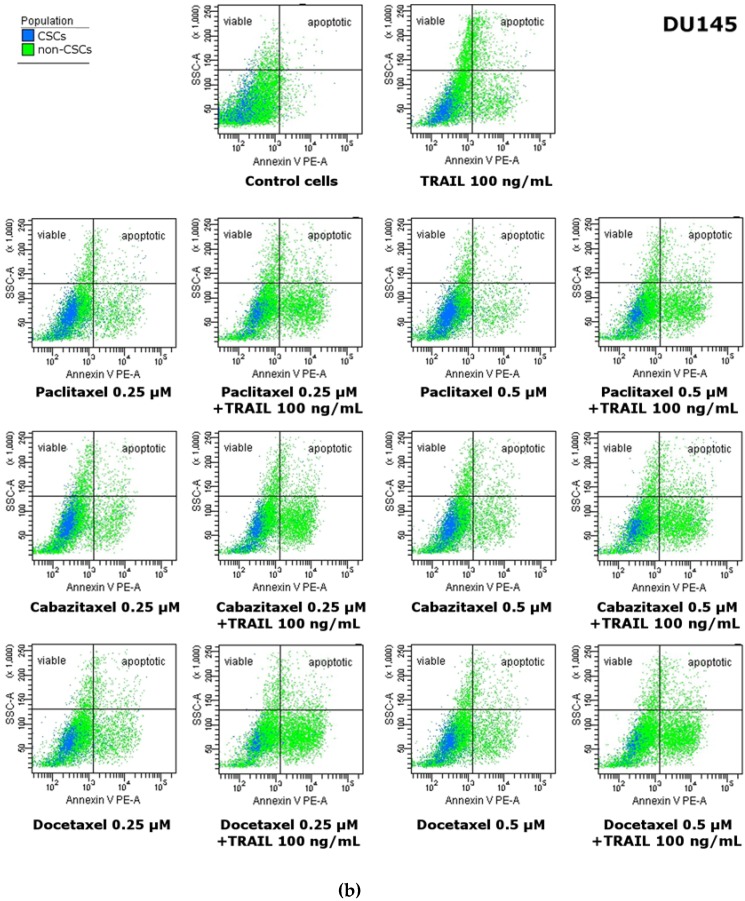
Apoptotic effect of TRAIL (100 ng/mL) in combination with paclitaxel, cabazitaxel and docetaxel on CSCs and non-CSCs subpopulations within: PC3 (**a**) and DU145 (**b**) prostate cancer cells. Cells were incubated with anti-CD24 FITC-labeled, anti-CD44 APC-labeled antibodies and Annexin V-PE labeled. CSCs marked as blue, non-CSCs marked as green. Viable cells are located on the left site of the dot plot and apoptotic cells on the right site of the dot plot.

Our results showed that CSCs in the PC3 and DU145 prostate cancer cells were significantly more resistant to apoptosis mediated by TRAIL compared to non-CSCs. Within PC3 cells the apoptotic effect of TRAIL at the concentration of 100 ng/mL following a 48 h incubation was 4.3% ± 0.9% of killed CSCs and 16.0% ± 2.7% of killed non-CSCs. In DU145 cells, apoptotic effect of 100 ng/mL TRAIL was 4.0% ± 1.7% of killed CSCs and 15.9% ± 1.7% of killed non-CSCs. Apoptotic effects analyzed by flow cytometry are presented in Figure 4 and Figure 5.

### 2.7. Apoptotic Activities of Paclitaxel, Cabazitaxel or Docetaxel within the Subpopulations in DU145 and PC3 Prostate Cancer Cell Lines

We have discovered that CSCs in the PC3 and DU145 prostate cancer cells were also more resistant to apoptosis mediated by taxanes compared to non-CSCs. Within PC3 cells the apoptotic effect of paclitaxel at the concentrations of 0.25 µM and 0.5 µM following a 48 h incubation was respectively 13.2% ± 1.3% and 13.2% ± 1.6% of killed CSCs whereas as much as 50.4% ± 8.2% and 54.8% ± 4.7% non-CSCs went apoptosis. Cabazitaxel at the concentrations of 0.25 µM and 0.5 µM killed respectively 10.7% ± 2.7% and 13.3% ± 3.3% CSCs while 41.6% ± 8.5% and 45.6% ± 9.0% non-CSCs went apoptosis. Eventually docetaxel at the concentrations of 0.25 µM and 0.5 µM killed respectively 13.35% ± 3.5% and 15.1% ± 1.6% CSCs whereas 52.6% ± 5.2% and 54.4% ± 5.4% non-CSCs went apoptosis.

Subsequently, DU145 cells appear to be more resistant then PC3 cells, however the most resistant were again CSCs. The apoptotic effect of paclitaxel on DU145 cells at the concentrations of 0.25 µM and 0.5 µM following a 48 h incubation was respectively 4.5% ± 0.7% and 4.6% ± 2.0% of killed CSCs whereas 16.1% ± 1.1% and 16.3% ± 2.8% non-CSCs went apoptosis. Cabazitaxel at the concentrations of 0.25 µM and 0.5 µM killed respectively 3.6% ± 1.1% and 4.8% ± 3.3% CSCs, while 15.7% ± 2.5% and 17.5% ± 2.3% non-CSCs went apoptosis. Ultimately, docetaxel at the concentrations of 0.25 µM and 0.5 µM killed, respectively, 4.3% ± 1.9% and 4.4% ± 1.1% CSCs, whereas 17.2% ± 2.1% and 16.8% ± 1.0% non-CSCs went apoptosis. The results of flow cytometric analysis are presented in Figure 4 and Figure 5.

### 2.8. Apoptotic Activities of TRAIL in combination with Paclitaxel, Cabazitaxel or Docetaxel within the Subpopulations in DU145 and PC3 Prostate Cancer Cell Lines

Our results showed that combined treatment of TRAIL and taxanes significantly enhanced the apoptotic effect on both subpopulations in PC3 prostate cancer cells compared to TRAIL or taxanes alone, whilst, in DU145 cells, the CSCs were resistant to apoptosis mediated by combination of taxanes and TRAIL. The co-treatment of TRAIL and taxanes augmented apoptosis effect only in non-CSCs subpopulation within DU145 cells. 

Within PC3 cells the co-treatment of cancer cells with TRAIL at the concentration of 100 ng/mL and paclitaxel at the concentrations of 0.25 µM and 0.5 µM increased the percentage of apoptotic cells, respectively, to 30.2% ± 5.7% and 34.2% ± 4.1% in CSCs subpopulation, and to 72.6% ± 7.1% and 75.9% ± 7.3% in non-CSCs subpopulation. After exposure to 100 ng/mL TRAIL and cabazitaxel at the concentrations of 0.25 µM and 0.5 µM, the apoptotic cells percentage was elevated to 29.6% ± 3.5% and 29.6% ± 6.1% within CSCs and to 71.6% ± 8.3% and 74.8% ± 9.2% in non-CSCs. Eventually, the co-treatment of 100 ng/mL TRAIL and docetaxel at the concentrations of 0.25 µM and 0.5 µM killed respectively 36.7% ± 4.2% and 35.8% ± 4.2% CSCs whereas 73.7% ± 4.8% and 81.3% ± 6.4% non-CSCs went apoptosis.

DU145 cells occur to be more resistant then PC3 cells, co-treatment with taxane and TRAIL has no significant impact on CSCs. Only non-CSCs appear to be more susceptible for simultaneous treatment with TRAIL and taxane. The apoptotic effect of 100 ng/mL TRAIL and paclitaxel at the concentrations of 0.25 µM and 0.5 µM following a 48 h incubation was respectively 6.3% ± 1.1% and 6.4% ± 1.7% of killed CSCs, whereas 28.3% ± 3.5% and 29.5% ± 3.3% non-CSCs went apoptosis. TRAIL and cabazitaxel at the concentrations of 0.25 µM and 0.5 µM killed, respectively, 3.9% ± 1.7% and 5.5% ± 0.9% CSCs, while 25.5% ± 4.2% and 26.0% ± 4.7% non-CSCs went apoptosis. Ultimately, co-treatment with TRAIL and docetaxel at the concentrations of 0.25 µM and 0.5 µM killed, respectively, 5.9% ± 1.5% and 4.8% ± 0.9% CSCs, whereas 30.7% ± 8.5% and 30.8% ± 3.6% non-CSCs went apoptosis. The results of flow cytometric analysis are presented in Figure 4 and Figure 5.

### 2.9. CD44+/CD24− Cancer Stem Cell Number in DU145 and PC3 Cell Lines after the Treatment of TRAIL and/or Paclitaxel, Cabazitaxel, Docetaxel

Our results showed that combined treatment with TRAIL and taxanes changed percentage of CSCs in both analyzed prostate cancer cell lines compared to TRAIL or taxanes alone. 

**Figure 6:**
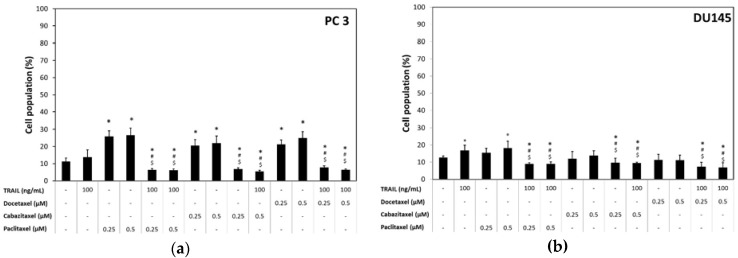
Effect of TRAIL (100 ng/mL) in combination with paclitaxel, cabazitaxel and docetaxel on CSCs number in DU145 and PC3 prostate cancer cells. * *p* < 0.05, significantly different from the respective control; # *p* < 0.05, significantly different from TRAIL alone; $ *p* < 0.05, significantly different from respective taxane alone. (**a**) CSCs number in PC3 prostate cancer cells after incubation with TRAIL (100 ng/mL) and/or paclitaxel, cabazitaxel, docetaxel; (**b**) CSCs number in DU145 prostate cancer cells after incubation with TRAIL (100 ng/mL) and/or paclitaxel, cabazitaxel, docetaxel.

In the control sample of PC3 cells, the CSCs subpopulation was 11.4% ± 1.9%, and after the treatment with 100 ng/mL TRAIL, 13.7% ± 4.4%. Exposure to 0.25 µM paclitaxel caused a significant increase to 25.5% ± 3.3%, and 0.5 µM paclitaxel to 26.6% ± 4.1% of CSCs subpopulation. However, the co-treatment of PC3 cancer cells with TRAIL at the concentration of 100 ng/mL and paclitaxel at the concentrations of 0.25 µM and 0.5 µM decreased the percentage of CSCs, respectively, to 6.3% ± 0.7% and 6.2% ± 0.7%. After exposure to cabazitaxel alone, at the concentrations of 0.25 µM and 0.5 µM, CSCs percentage increased, respectively, to 20.6% ± 3.3% and 22.0% ± 4.1%. The co-treatment with TRAIL at the concentration of 100 ng/mL and cabazitaxel at the concentrations of 0.25 µM and 0.5 µM, decreased the percentage of CSCs, respectively, to 6.8% ± 0.7% and 5.5% ± 0.6%. Eventually, the treatment of docetaxel at the concentrations of 0.25 µM and 0.5 µM also caused a significant increase in CSCs number, respectively, to 21.3% ± 2.5% and 25.0% ± 3.5%. The co-treatment with TRAIL at the concentration of 100 ng/mL and docetaxel at the concentrations of 0.25 µM and 0.5 µM decreased the percentage of CSCs, respectively, to 7.9% ± 0.9% and 6.5% ± 0.5%. The described effect of TRAIL and/or paclitaxel, cabazitaxel and docetaxel on CSCs number in PC3 prostate cancer cells is presented in Figure 6a.

In the DU145 cell line, the CSCs subpopulation was 12.6% ± 0.9% and after the treatment with 100 ng/mL TRAIL, 16.8% ± 3.3%. Exposure to 0.25 µM and 0.5 µM paclitaxel changed the CSCs number, respectively, to 15.4% ± 2.7%, and 18.1% ± 4.1%. On the other hand, the co-treatment of DU145 cancer cells with TRAIL at the concentration of 100 ng/mL and paclitaxel at the concentrations of 0.25 µM and 0.5 µM decreased the percentage of CSCs to 9.1% ± 0.6% and 9.0% ± 1.0%, respectively. Cabazitaxel alone, at the concentrations of 0.25 µM and 0.5 µM, changed CSCs percentage respectively to 12.0% ± 4.1% and 13.8% ± 2.8%. However, the co-treatment with TRAIL at the concentration of 100 ng/mL and cabazitaxel at the concentrations of 0.25 µM and 0.5 µM, decreased the percentage of CSCs, respectively, to 9.7% ± 2.6% and 9.5% ± 0.5%. Eventually, the treatment of docetaxel at the concentrations 0.25 µM and 0.5 µM caused a slight decrease in CSCs number, respectively to 11.3% ± 3.3% and 11.1% ± 2.9%. Interestingly, the co-treatment with TRAIL at the concentration of 100 ng/mL and docetaxel at the concentrations of 0.25 µM and 0.5 µM significantly decreased the percentage of CSCs, respectively, to 7.2% ± 2.5% and 6.95% ± 2.6%. The described effect of TRAIL and/or paclitaxel, cabazitaxel and docetaxel on CSCs number in DU145 prostate cancer cells is presented in Figure 6b.

### 2.10. Expression of TRAIL-Receptors (R1, R2, R3 and R4) on the Surface of PC3 and DU145 Prostate Cancer Cells

**Figure 7:**
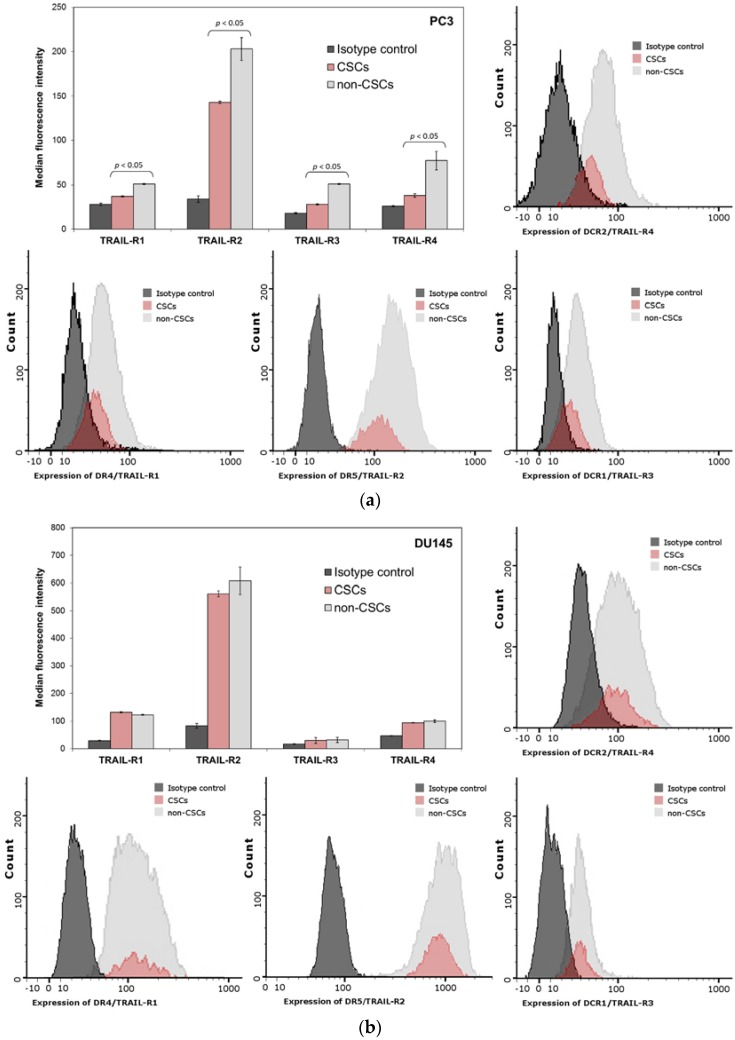
Expression of TRAIL-Receptors (R1, R2, R3 and R4) on the Surface of CSCs and non-CSCs subpopulations within: PC3 (**a**); and DU145 (**b**) prostate cancer cells. The dark grey histogram shows isotype control, red corresponds to CSCs, and light grey histogram shows non-CSCs.

The sensitivity of cancer cell to TRAIL is connected with the expression of death receptors TRAIL-R1/DR4 and TRAIL-R2/DR5. We have analyzed by flow cytometry the expression of TRAIL-R1/DR4 and TRAIL-R2/DR5 on the surface of CSCs and compared it to non-CSCs subpopulation. In PC3 and DU145 cells, the fluorescence signal corresponding to TRAIL-R1 expression was very dim. In DU145 cells, our results demonstrated no significant difference between the populations of CSCs and non-CSCs, while, in PC3 cells, there was only slight difference. On the other hand, the signal corresponding to TRAIL-R2 expression was much brighter. Furthermore, we have detected that TRAIL-R2 expression in non-CSCs subpopulation was significantly higher than in CSCs for the PC3 cancer cells. The results of the experiment are presented in Figure 7. 

We have also analyzed the expression of decoy receptors TRAIL-R3 and TRAIL-R4 on the surface of CSCs and the non-CSCs subpopulation. The expression of decoy receptors in PC3 and DU145 cancer cells was very low and probably irrelevant.

### 2.11. Effects of Paclitaxel, Cabazitaxel or Docetaxel on TRAIL-Receptors (R1, R2) Expression on the Surface of PC3 and DU145 Prostate Cancer Cells

**Figure 8:**
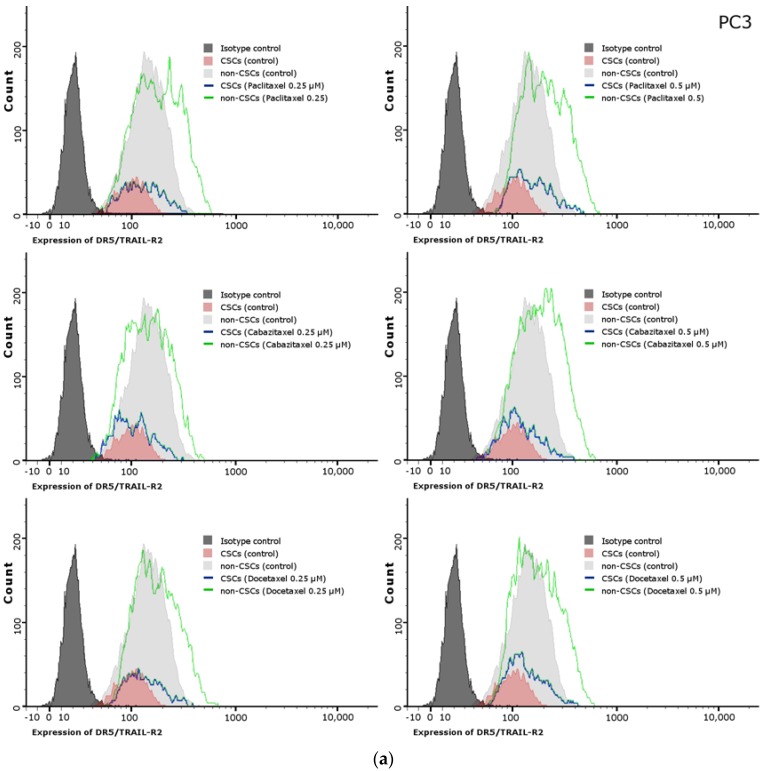

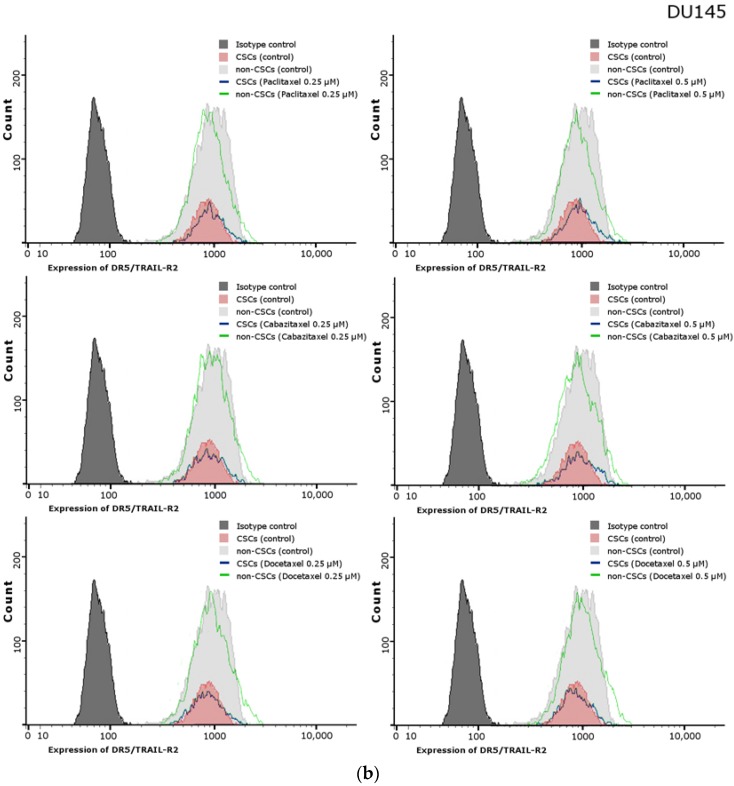
Effects of paclitaxel, cabazitaxel or docetaxel on expression of TRAIL-R2 on the surface of CSCs and non-CSCs subpopulations within: PC3 (**a**); and DU145 (**b**) prostate cancer cells. The dark grey histogram shows isotype control, red corresponds to CSCs, light grey histogram shows non-CSCs, blue line shows CSCs after treatment with adequate taxane, and green line corresponds non-CSCs treated with adequate taxane.

We have shown that paclitaxel, cabazitaxel and docetaxel enhance TRAIL-mediated apoptosis, therefore the next step was to determine if this compounds increase the expression of death domain-containing transmembrane receptors: TRAIL-R1 (DR4) and TRAIL-R2 (DR5). In PC3 and DU145 cells, flow cytometry was employed to show that the fluorescence signal corresponding to TRAIL-R1 expression was again very dim. The median values of fluorescence intensity have not been changed after the exposure to analyzed taxanes (data not shown). On the other hand, the TRAIL-R2 (DR5) is upregulated on the surface of PC3 cells after the treatment with taxanes. Both subpopulations, CSCs and non-CSCs, have elevated expression of this receptor following the treatment with paclitaxel, cabazitaxel and docetaxel. However, on the surface of DU145, the expression of TRAIL-R2 (DR5) is almost unchanged after the exposition to paclitaxel, cabazitaxel and docetaxel. The described effect of paclitaxel, cabazitaxel and docetaxel on TRAIL-R2 expression on the surface of PC3 and DU145 prostate cancer cells is presented in Figure 8.

## 3. Discussion

Prostate cancer stem cells (CSCs) are a small subpopulation of cancer cells, which possess stem-like properties, such as the ability to self-renew, and are capable of driving tumor growth and metastasis [4,35]. These cells are also more resistant to hormonotherapy, radiotherapy and chemotherapy than differentiated daughter cells. Numerous studies and clinical evidence suggest an increased CSCs in tumor mass may contribute to the failure of the therapy. Current therapeutic protocols often lead to the disease progression, because they target the bulk of fast growing cancer cells but not undifferentiated, slow cycling cancer stem cells [36,37,38].

Many potential stem cells markers have been identified in prostate, however the CD44+/CD24− cells have been associated with the prostate cancer stem cells which possess the ability to grow as nonadherent spheres in serum replacement medium and a potential to form tumors in NOD/SCID mice. It has been shown that CD44 expression can be connected with the increased clonogenic and metastatic potential [9,14]. 

New therapeutics introduced to prostate cancer treatment have greatly improved the overall survival of the patients, however the relapse of chemoresistant tumor remains the major problem. Generally, most therapeutics only target the dividing tumor cells, therefore the CSCs population remain intact. Consequently, the expansion of CSCs population results in rising drug resistance among the cells constituting the tumor mass [7,8,30].

Acquired resistance to taxane based chemotherapy remains a major consideration for patients who show an initial therapeutic benefits from taxane treatment. The stem-cells directed therapy could sensitize these cells to anticancer drugs [39,40].

TRAIL (tumor necrosis factor-related apoptosis inducing ligand) is a promising anti-cancer agent because of its selectivity. It induces apoptosis in cancer cells but not in normal cells. In our research, we examined if treatment with one of the three different taxanes can enhance apoptosis induced by TRAIL. We observed that paclitaxel, cabazitaxel and docetaxel in combination with TRAIL effectively induce apoptosis in PC3 cells. In DU145 cells, the apoptosis was less intense but also significant. The apoptotic cell death induced by taxanes and TRAIL alone was significantly lower in both cell lines. Our results correspond to other previous observations that chemotherapeutic agents can enhance TRAIL-induced apoptosis in human cancers such as breast cancer [41,42], squamous carcinoma [43], colon cancer [44], renal carcinoma, bladder cancer, lung cancer and prostate cancer [45,46]. Taxanes, such as paclitaxel, in combination with TRAIL have been shown to induce apoptosis of 86M1 small cell lung cancer cells [47], resistant gastric cancer cells [48] and PC3, DU145 and LNCaP prostate cancer cells [49]. Moreover, pretreatment of docetaxel have been shown to enhance apoptosis mediated by TRAIL in prostate cancer cells [50].

Furthermore, we examined using flow cytometry and test with Annexin V the apoptotic cell death in two subopulations, CSCs and non-CSCs, gated among the PC3 and DU145 cells. In both cell lines, CSCs were much more resistant to apoptotic effect of TRAIL, paclitaxel, cabazitaxel or docetaxel used alone. The co-treatment with taxanes and TRAIL enhanced the apoptotic cell death in both subpopulations (CSCs and non-CSCs) in PC3 prostate cancer cells. While in DU145 cells the co-treatment with taxanes and TRAIL enhanced significantly apoptosis only in non-CSCs subpopulation. 

We also examined the incidence of CSCs in PC3 and DU145 cancer stem cells. In both cell lines, CSCs subpopulation comprised about 10–13% of all cells. Interestingly, PC3 and DU145 cells after the treatment with each taxane alone, have a larger proportion of CSCs (up to 26% in PC3 and up to 18% in DU145), whereas the treatment with paclitaxel, cabazitaxel or docetaxel in combination with TRAIL resulted in reduction of CSCs frequency to the level of 5% in PC3 cells and 7% in DU145 cells. This result may indicate that paclitaxel, cabazitaxel and docetaxel kills preferentially the non-CSCs cells whereas CSCs subpopulation is more resistant. However using a combination of agents: paclitaxel, cabazitaxel or docetaxel and TRAIL have an opposite effect—the number of CSCs in both cell lines were reduced. These data suggest that co-treatment with taxane and TRAIL can enhance the CSCs subpopulation cell death. Therefore, the percentage of CSCs decreases.

Cells with stem-like properties have been studied recently and associated with unfavorable prognosis and aggressive disease. In breast carcinoma CD44 positive and CD24 negative cells have been found in distant metastasis, therefore this phenotype is connected with a relapse after surgical resection [51,52]. PC3 and DU145 cell lines have been derived from the prostate cancer patients with a distinct metastasis (to the lymph node and the brain). This explains the occurrence of the CD44+/CD24− cells in both cell lines.

Conventional therapies in the cancer treatment kill the rapidly dividing cells resulting in tumor withdrawal. However, the CSCs which are more quiescent and resistant to anti-cancer agents, differentiate into new tumor cells and are responsible for tumor relapse. CSCs express high levels of ATP-binding cassette drug transporters [53,54], cell adhesion molecules (CD44) [55], or cell survival proteins which are responsible for their chemoresistance [56]. Thus far, there have been only a few approaches to overcome this resistance and improve the efficiency of the chemotherapeutic drugs against CSCs population in the breast cancer, prostate cancer and ovary cancer cells [57,58].

Interestingly, even naturally occurring compounds have been found to enhance the efficacy of chemotherapeutics. Green tea and a flavonoid quercetin enhanced the efficacy of docetaxel in LAPC-4-AI and PC-3 prostate cancer cells. Furthermore, the combination of this compound and docetaxel inhibited tumor cell invasion, colony formation and decreased the percentage of CSCs [59].

Taxanes are highly active anticancer drugs which have been shown to trigger apoptosis not only by intrinsic (mitochondrial) pathway but also extrinsic pathway (by induction of death receptors expression) [47,48,49,60]. It was discovered before, that treatment of prostate cancer cells with paclitaxel and TRAIL resulted in greater processing of caspase-8, Bid, procaspase-9 and caspase-3, resulting in engagement of the mitochondrial pathway to apoptosis [49]. Hunter et al. discovered, that in non-small cell lung cancer cells treated with paclitaxel and TRAIL, the expression of pro-survival protein Bcl-xl, was down regulated. They also found out that after the treatment with paclitaxel and TRAIL, apoptosis inducing factor (AIF) was cleaved into mature form, then left the mitochondria and was translocated to the nucleus where it was able to initiate DNA degradation [47]. Finally, Li et al. showed that inhibition of MAPK was involved in paclitaxel-mediated sensitization to TRAIL in resistant gastric cancer cells [48]. Moreover, also docetaxel was shown to enhance TRAIL-induced apoptosis through caspase-8, caspase-9 and caspase-3 activation in different prostate cancer cell lines [50].

We have discovered that paclitaxel, cabazitaxel and docetaxel enhance TRAIL-mediated apoptosis especially in non-CSCs population. Next, we tried to determine if those compounds increase the expression of death receptors: TRAIL-R1 (DR4) and TRAIL-R2 (DR5). TRAIL-R1 expression was very low and did not change after taxanes treatment in both cell lines. Nevertheless, we discovered that TRAIL-R2 (DR5) expression was increased on the surface of PC3 cells treated with taxanes. In addition, we have observed that on the surface of CSCs TRAIL-R2 expression was lower compared to non-CSCs population. Furthermore, both subpopulations, CSCs and non-CSCs have elevated expression of this receptor following the treatment with paclitaxel, cabazitaxel and docetaxel in PC3 cells. The level of TRAIL-R2 expression in DU145 cells remained unchanged after treatment with taxanes, which could explain their resistance. The mechanism by which taxanes sensitizes prostate cancer cells to TRAIL-induced apoptosis is not clear and a further study are required to explain how these chemotherapeutics affect the apoptotic signaling pathways.

It has been demonstrated that chemotherapeutic agents can have an influence on TRAIL-R2-mediated apoptosis and cytotoxicity in various human solid cancer cells. It was proven that low concentrations of doxorubicin significantly increased TRAIL-R2 expression in human prostate, bladder, and lung cancer cells [46]. It was discovered that in human gastric cancer paclitaxel markedly enhanced TRAIL-induced apoptosis by activation of mitochondrial pathway, upregulation of TRAIL receptors and downregulation of anti-apoptotic proteins [48]. Eventually, it was revealed that docetaxel enhances TRAIL-mediated apoptosis due to upregulation of TRAIL-R2 in melanoma cells [61]. However, there is no published paper regarding CSCs population in prostate cancer and TRAIL-mediated apoptosis.

Presently, we can only speculate about mechanisms of apoptosis activation by different taxane derivatives and their cooperation with TRAIL. There is not enough evidence yet, and still many questions remain to be answered to understand the CSCs role in the chemoresistance of prostate cancer. 

## 4. Materials and Methods 

### 4.1. Cell Lines

Human prostate cancer DU145 and PC3 cell lines was purchased from DSMZ (Deutsche Sammlung von Mikroorganismen und Zellkulturen, Braunschweig, Germany). The DU145 cells were grown in RPMI-1640 medium. The PC3 cells were maintained in RPMI-1640 with the addition of Ham’s medium. The media were supplemented with 10% heat-inactivated fetal bovine serum, 4 mM L-glutamine, 100 U/mL penicillin and 100 µg/mL streptomycin. The cells were incubated at 37 ℃ in a humidified atmosphere of 5% CO_2_. Reagents for cell culture were purchased from ATCC (American Type Culture Collection, Manassas, VA, USA).

### 4.2. Reagents

Soluble recombinant human TRAIL (rhsTRAIL) was obtained from PeproTech Inc. (Rocky Hill, NJ, USA). Semisynthetic paclitaxel and docetaxel were purchased from Sigma Aldrich Chemical Company (St. Louis, MO, USA). The purity of paclitaxel and docetaxel was ≥97% (HPLC). Cabazitaxel was purchased from LC Laboratories (Woburn, MA, USA). The purity of cabazitaxel was ≥99% (HPLC). Tested compounds were dissolved in DMSO (dimethyl sulfoxide) to obtain the working concentrations. 

### 4.3. Detection of Apoptotic and Necrotic Cell Death by Flow Cytometry

Apoptosis was measured by flow cytometry using the Apoptosis-PE Kit with Annexin V (Becton Dickinson Biosciences, San Jose, CA, USA). Prostate cancer cells DU145 and PC3 (5 × 10^4^/mL) were seeded in 24-well plates for 24 h and then exposed to TRAIL in concentration of 100 ng/mL and/or one of the taxanes (paclitaxel, docetaxel or cabazitaxel) for 48 h. After this time the cells were harvested using trypsin and ethylenediaminetetracetic acid (EDTA) and then washed twice with phosphate-buffered saline (PBS) solution and resuspended in 1× Binding Buffer (100 µL). Then the cell suspension was incubated with Annexin V-PE (5 µL) and 7-AAD (5 µL) for 10 min at 4℃ in the dark. After this time 400 µL of 1× Binding Buffer was added to each tube. The cells were analyzed by flow cytometry (LSR II, Becton Dickinson Biosciences, San Jose, CA, USA) within 1 h.

### 4.4. Lactate Dehydrogenase Release Assay

Lactate dehydrogenase (LDH) activity was measured in culture supernatants with an enzymatic assay, based on the conversion of tetrazolium salt into a red formazan product. Reagents were purchased from Roche Diagnostics GmbH (Mannheim, Germany). DU145 and PC3 cells were treated with various concentrations of one of the taxanes (paclitaxel, docetaxel or cabazitaxel) for the specified period of time. Then, supernatant was removed, and the LDH release from the cells to the culture medium was measured by the spectrophotometer at 490 nm. Additionally, control cells were treated with 1% Triton X-100 for 10 min (room temperature) to obtain maximal LDH release. The percentage of the necrotic cells was calculated according to a formula:

% Cytotoxicity = (Compound-treated LDH Release − Spontaneous LDH Release/Maximal Release − Spontaneous LDH Release) × 100(1)

### 4.5. Detection of CD44+/CD24− Cancer Stem Cells by Flow Cytometry

CD44+/CD24− subpopulation of cancer stem cells was labeled using anti-CD24-FITC and anti-CD44-APC monoclonal antibodies (Becton Dickinson Biosciences, San Jose, CA, USA). Prostate cancer cells DU145 and PC3 (5×10^4^/mL) were seeded in 24-well plates for 24 h and then exposed to TRAIL in concentration of 100 ng/mL and/or one of the taxanes (paclitaxel, docetaxel or cabazitaxel) for 48 h. After this time the cells were harvested using trypsin and ethylenediaminetetracetic acid (EDTA) and then washed twice with phosphate-buffered saline (PBS) solution and resuspended in PharmingenStain Buffer with BSA (100 µL) (Becton Dickinson Biosciences, San Jose, CA, USA). Then the cell suspension was incubated with anti-CD24-FITC (20 µL) and anti-CD44-APC (20 µL) monoclonal antibodies for 20 minutes in the dark. Cells in separate tubes treated with mouse IgG1 APC and IgG2a FITC monoclonal antibody constituted the isotype controls (Becton Dickinson Biosciences, San Jose, CA, USA). After incubation, 400 µL of PharmingenStain Buffer (BSA) was added to each tube. Finally, the cells were analyzed by flow cytometry (LSR II, Becton Dickinson Biosciences, San Jose, CA, USA) within 1 h.

### 4.6. Detection of Apoptotic Cell Death by Flow Cytometry in the Subpopulations of Cells in DU145 and PC3 Prostate Cancer Cell Lines 

Apoptosis was measured by flow cytometry using the Apoptosis-PE Kit with Annexin V. Subpopulation of cancer stem cells was labeled as CD44+/CD24− using anti-CD24-FITC and anti-CD44-APC monoclonal antibodies (Becton Dickinson Biosciences, San Jose, CA, USA). Prostate cancer cells DU145 and PC3 (5 × 10^4^/mL) were seeded in 24-well plates for 24 h and then exposed to TRAIL in concentration of 100 ng/mL and/or one of the taxanes (paclitaxel, docetaxel or cabazitaxel) for 48 h. After this time the cells were harvested using trypsin and ethylenediaminetetracetic acid (EDTA), then washed twice with phosphate-buffered saline (PBS) solution and resuspended in 1× Binding Buffer (100 µL). The cell suspension was incubated with Annexin V-PE (5 µL), anti-CD24-FITC (20 µL) and anti-CD44-APC (20 µL) monoclonal antibodies for 20 min at 4 ℃ in the dark. After this time 400 µL of 1× Binding Buffer was added to each tube. The cells were analyzed by flow cytometry (LSR II, Becton Dickinson Biosciences, San Jose, CA, USA) within 1 h.

### 4.7. Analysis of Death Receptor Expression on the Cancer Cell Surface by Flow Cytometry 

The expression of four death receptors (DR4/TRAIL R1, DR5/TRAIL R2, TRAIL R3 and TRAIL R4) was determined using appropriate monoclonal antibodies conjugated with phycoerythrin (PE) or peridinin chlorophyll (PerCP) purchased from R&D Systems (Minneapolis, MA, USA). Prostate cancer cells DU145 and PC3 (5 × 10^4^/mL) were seeded in 24-well plates for 24 h and then exposed to various concentrations of the taxanes (paclitaxel, docetaxel or cabazitaxel) for 48 h. After this time the cells were harvested using Accutase Solution (PAN-Biotech GmbH, Aidenbach, Germany). The cells were further incubated in medium for 6 h on a rocker platform to enable regeneration of the receptors. The cells were washed with PBS (with 0.5% BSA) and then Fc-blocked (1µg IgG/10^6^ cells) for 15 min at room temperature. After this time an appropriate conjugated antibody was added (10 µL of anti-TRAIL R-1, anti-TRAIL R-2, anti-TRAIL R-3 or anti-TRAIL R-4 for 4 × 10^5^ cells). Then the cells were incubated in 4 ℃ for 20 min. After this time to each tube additional antibodies were added: anti-CD24-FITC and anti-CD44-APC (Becton Dickinson Biosciences, San Jose, CA, USA). The cells were incubated in 4 ℃ for another 20 min. After the incubation the cells were washed with PBS and finally analyzed by flow cytometry. Cells in separate tubes treated with appropriate mouse IgG (IgG1, IgG2a or IgG2b) monoclonal antibody conjugated with PE, PerCP, APC or FITC constituted the isotype control. Anti-TRAIL-R isotype controls were purchased from R&D Systems (Minneapolis, MA, USA) and anti-CD24 and anti-CD44 isotype controls from Becton Dickinson Biosciences (San Jose, CA, USA).

### 4.8. Statistical analysis

The results are presented as means ± SD (*n* = 6). Statistical significance was evaluated using ANOVA or Student’s *t*-test. *p* < 0.05 was considered significant. For statistical calculation, StatSoft Statistic version 12 or Microsoft Excel 2010 was used.

## Abbreviations

CSC	cancer stem cell
TRAIL	tumor necrosis factor-related apoptosis-inducing ligand
PE	phycoerythrin
TRAIL-R	receptor of tumor necrosis factor-related apoptosis-inducing ligand
NOD/SCID	non-obese diabetic/severe combined immunodeficiency
TNF	tumor necrosis factor
DR	death receptor
CRPC	castration-resistant prostate cancer
FDA	food and drug administration
SD	standard deviation
LDH	lactate dehydrogenase
7-AAD	7-aminoactinomycin D
non-CSC	non-cancer stem cell
FITC	fluorescein isothiocyanate
APC	allophycocyanin
DCR	decoy receptor
ATP	adenosine triphosphate
AIF	apoptosis inducing factor
MAPK	mitogen-activated protein kinase
DSMZ	Deutsche Sammlung von Mikroorganismen und Zellkulturen
RPMI	Roswell Park Memorial Institute medium
ATCC	American Type Culture Collection
DMSO	dimethyl sulfoxide
EDTA	ethylenediaminetetracetic acid
PBS	phosphate-buffered saline
PerCP	peridinin chlorophyll
ANOVA	analysis of variance
